# The Sistine Chapel and clothesline signs: a tale of two arteries

**DOI:** 10.1055/s-0042-1758395

**Published:** 2022-12-28

**Authors:** Léo Coutinho, João Matheus Tussolini Marcon, Ellen Riedi Oliveira, Camila Emi Fujiwara Murakami, Jessica Castro Silva, Juliano André Muzzio, Viviane Hiroki Flumignan Zetola, Marcos Christiano Lange, Carlos Alberto Engelhorn, Paulo Cesar Evaristo Souza, Hélio Afonso Ghizoni Teive

**Affiliations:** 1 Universidade Federal do Paraná, Programa de Pós-Graduação em Medicina Interna e Ciências da Saúde, Grupo de Doenças Neurológicas, Curitiba PR, Brazil.; 2 Universidade Federal do Paraná, Curso de Medicina, Curitiba PR, Brazil.; 3 Universidade Federal do Paraná, Serviço de Neurologia, Unidade Cerebrovascular, Curitiba PR, Brazil.; 4 Angiolab, Curitiba PR, Brazil.; 5 Universidade Federal do Paraná, Serviço de Neurorradiologia, Curitiba PR, Brazil.; 6 Universidade Federal do Paraná, Serviço de Neurologia, Unidade de Distúrbios de Movimento, Curitiba PR, Brazil.

**Figure 1:**
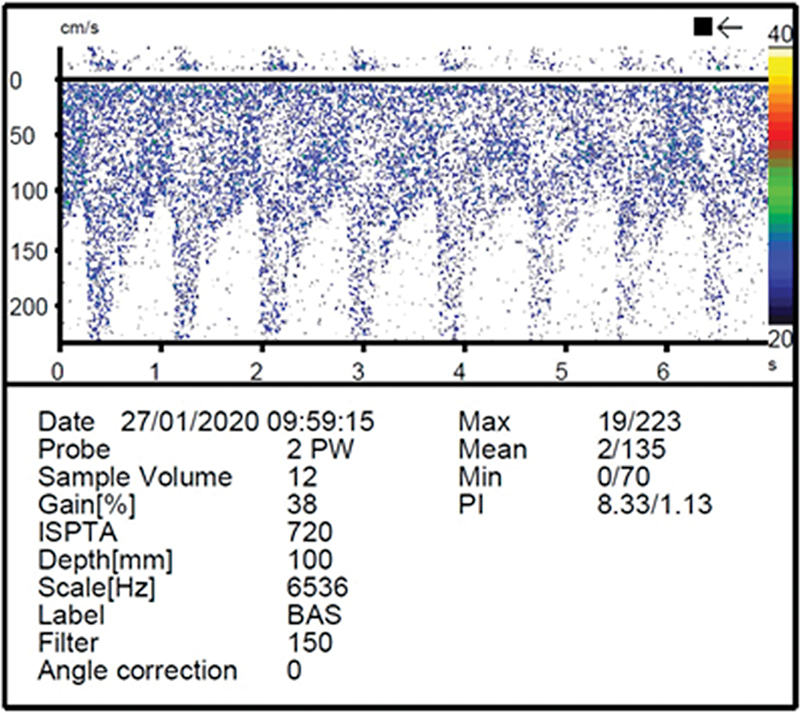
Transcranial Doppler show signs of segmental increase in flow velocity in the proximal basilar artery, compatible with basilar artery stenosis.

**Figure 2:**
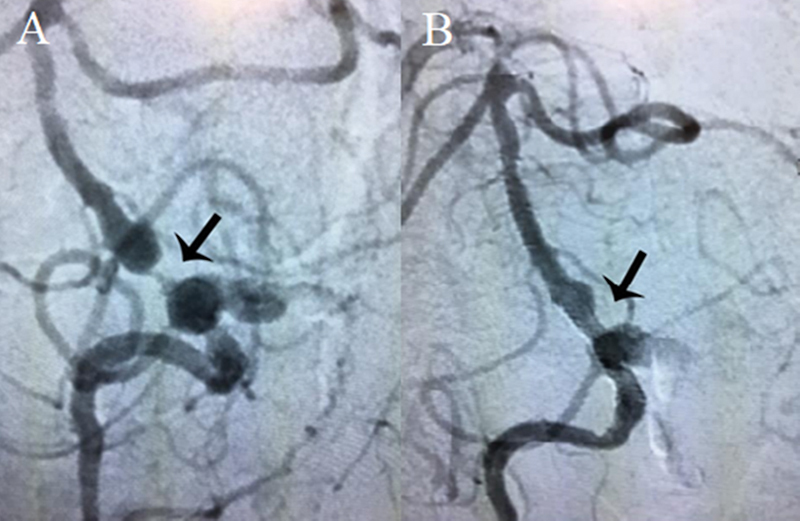
Brain arteriography showing severe stenosis in the proximal basilar artery **(A)** . Postangioplasty control with stent in the proximal basilar artery **(B)** .

**Figure 3:**
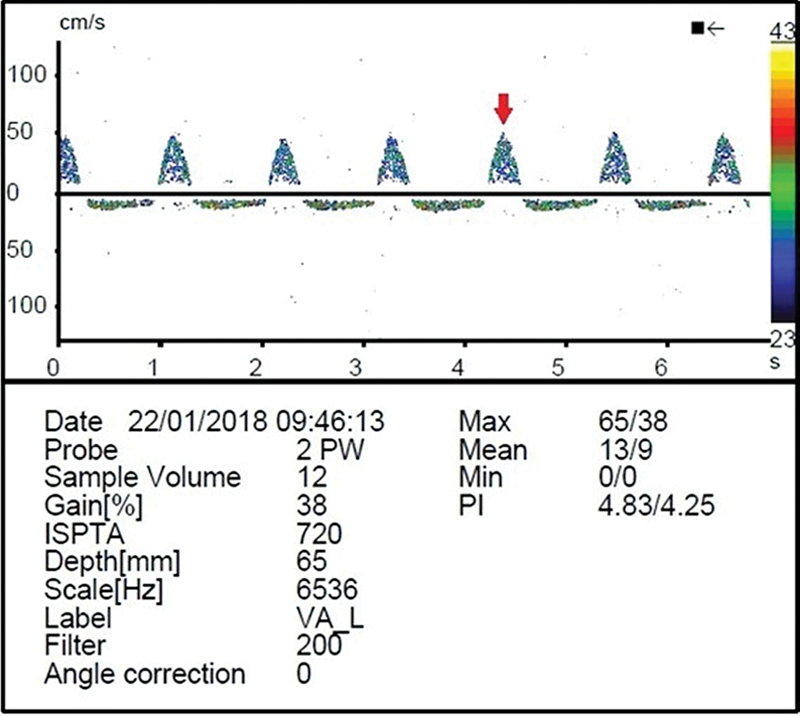
Systolic flow reversal in the left vertebral artery at transcranial doppler ultrasound (arrow), compatible with grade 2 subclavian steal syndrome (intermittent or partial).

**Figure 4:**
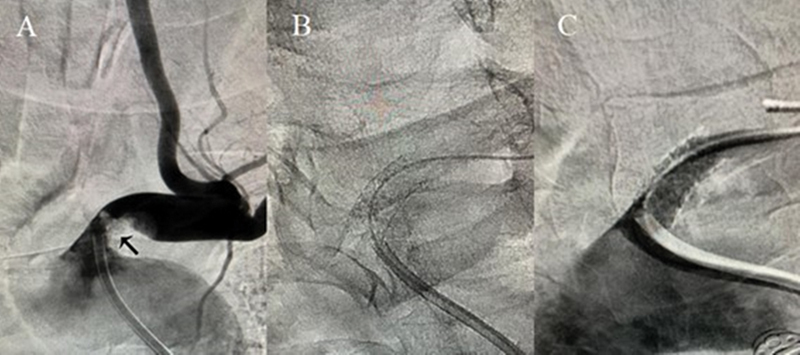
Cervical arteriography demonstrating a critical lesion in the origin of the left subclavian artery, promoting subclavian steal syndrome **(A)** . Cervical arteriography after subclavian artery angioplasty with stent positioning **(B) (C)** .

An 83-year-old woman presented sudden vertigo, drop attack, and transient dysarthria after head hyperextension to see the Sistine Chapel ceiling, in the Vatican. Transcranial doppler ultrasound ( Figure 1 ) suggested proximal basilar stenosis, confirmed by arteriography ( Figure 2 ). Case 2: A 77-year-old woman presented a 1-year history of transient vertigo after hanging clothes on a clothesline. Transcranial doppler ultrasound ( Figure 3 ) revealed left subclavian artery steal phenomenon secondary to proximal subclavian artery stenosis, confirmed by arteriography ( Figure 4 ). They received stenting and dual antiplatelet therapy. Transient ischemic symptomatology triggered by head/neck and arm movements demands vertebrobasilar and subclavian evaluation. 1 2 3
